# Editorial Briefs

**Published:** 1886-11-20

**Authors:** 


### EDITORIALS AND MISCELLANEOUS.

### EDITORIAL BREVITIES.

CORRECTION.—In Dr. Durham's article, in our October number, in last paragraph but one, the sentence "In about six months," should read : ***For six months afterwards***, etc.

CLINICAL LECTURES from the Professors of the Southern Medical College, and occasionally from the Post-Graduate Medical School of New York, will be published in the RECORD from time to time.

COCAINE ADDICTION.—Dr. J. B. Mattison, 314 State street, Brooklyn, N.Y., is anxious to hear of any cases of cocaine addiction. Parties knowing of such cases are requested to write to him at the above address.

PEPTO-QUININE PILLS.—We are in receipt of a beautiful sample of these pills, in the shape of compressed tablets. The combination we regard as valuable, and likely to be acceptable to many cases where the quinine alone would not agree. We shall give them a trial in practice. We thank the proprietors for the sample sent us. They are prepared by the Pennsylyania Chemical Manufacturing Company, Philadelphia.

DR. JAMES WAKELY, the renowned editor of the *London Lancet*, died recently of a cancerous affection. On his death bed, he requested the following confession of faith to be put into his obituary notice : "Feeling my deep responsibility to God for the position in which, in His providence, He placed me, I desire to acknowlege the comfort derived during my sickness from a lively faith in our Lord Jesus Christ, and that I die in the sure hope of a glorious resurrection."

RIO CHEMICAL COMPANY.—We are in receipt of beautiful samples from the Rio Chemical Company, of Saint Louis, Mo. Among these we mention as especially good, the Aletris Cordial, a uterine tonic and restorative ; Celerina, an excellent nervine ; Acid Mannate, highly recommended as a mild laxative, adapted especially to women in child-bed ; Pinus Canadensis, a valuable agent in chronic diseases of the mucous membranes, and admirable for the removal of morbid discharges of every kind.

CAUTION.—Battle & Co. complain that some one is seeking to counterfeit their Bromidia. Dr. George Springer writes them from Ohio that "In the case of Insomnia which I reported to you in May last, and wherein it required seven drachm-doses (hourly, 1 drachm) to produce sleep by Bromidia bought at pharmacy in Findlay—it required but 1 drachm, repeated in one hour, to produce a good night's rest, of the sample bottle you sent me. I also use the Bromidia (Battle & Co.) with the best results in Cholera Infantum, and in Hysteria. Am satisfied that the article bought at Findlay was spurious."

SPECIAL NEW YORK CORRESPONDENT.—David C. Bryan, M. D., Demonstrator of the Normal and Pathological Anatomy of the Nervous System in the New York Post-Graduate Medical School, will hereafter act as Special Correspondent for the RECORD from the city of New York. By virtue of this arrangement, our readers may expect, occasionally, something both interesting and practical from the great city of New York.

PHARMACY IN ATLANTA.—We have been favored with beautiful samples of drug preparations put up in Atlanta by the enterprising house of Curry, Jacobs & Co., formerly *Jacobs' Pharmacy*, corner of Peachtree and Marietta streets. The neatness and purity of the preparations compare favorably with those of our best Northern manufacturers in the drug line. It is gratifying to note the evidences of the continued growth and development of our city in all departments of business, and to be able to say that we have here business talent and enterprise that places us alongside of the most prosperous cities of the Union.

DEATH OF DR. ATTAWAY.—We are pained to learn of the death of Dr. Oscar Attaway, of Roswell, Ga., which occurred on November 5, 1886, in the thirtieth year of his age. Dr. Attaway was a graduate of the Southern Medical College of Atlanta—Class of 1884–'85. He held a high position in the examination, and was warmly esteemed by the Faculty. He was a close student, devoted to the profession, and loved the practice of Medicine for the good that it enabled him to do rather than for the honor it conferred. During his brief career, though feeble in health, he rapidly grew in public confidence, and gave promise of a high reputation in the profession. He was an upright Christian gentleman, and died in the full triumph of faith. He was called, and has cheerfully gone to the other side.

NEW DEPARTURE IN BOOKS.—The publishing house of G. S. Davis, Detroit, Mich., has made a new departure in Medical publications, by which books are to be furnished in " short, practical treatises, prepared by well known authors, containing the gist of what they have to say," especially regarding the treatment of diseases commonly met with. Sold at a small price. They have commenced a series of twelve works upon this plan—the whole series to cost only $2.50, and a separate volume to be sold at twenty-five cents. Each of the series will be reviewed in this journal as they are received.

THE MEDICAL NEWS VISITING LIST FOR 1887, containing Calendar for two years ; Obstetric diagrams ; Scheme of Dentition ; Tables of Weights and Measures and comparative scales ; Instructions for examining the urine ; List of Disinfectants ; Table of Eruptive Fevers ; Lists of new remedies and remedies not generally used ; Incompatibles, Poisons and Antidotes ; Artificial Respiration ; Table of Doses, prepared to accord with the last revision of the U.S. Pharmacopœia, an extended table of Diseases and their Remedies, and directions for Ligation of Arteries ; Blanks for all records of practice and Erasable Tablet. Handsomely bound in limp Morocco, with tuck, pocket, pencil, rubber, and catheter scale. Lea Brothers & Co., publishers, Philadelphia, Pa. Price, $1.25.

